# Technology-Based Music Interventions to Reduce Anxiety and Pain Among Patients Undergoing Surgery or Procedures: Systematic Review of the Literature

**DOI:** 10.2196/48802

**Published:** 2024-07-08

**Authors:** Sunghee Park, Sohye Lee, Sheri Howard, Jeeseon Yi

**Affiliations:** 1 Nursing Department Ajou University Hospital Suwon Republic of Korea; 2 College of Nursing Ajou University Suwon Republic of Korea; 3 Loewenberg College of Nursing The University of Memphis Memphis, TN United States; 4 College of Nursing & Sustainable Health Research Institute Gyeongsang National University Jinju Republic of Korea

**Keywords:** technology, music intervention, anxiety, pain

## Abstract

**Background:**

Hospitalized patients undergoing surgery or procedures may experience negative symptoms. Music is a nonpharmacological complementary approach and is used as an intervention to reduce anxiety, stress, and pain in these patients. Recently, music has been used conveniently in clinical situations with technology devices, and the mode of providing music is an important factor in technology-based music interventions. However, many reviews have focused only on the effectiveness of music interventions.

**Objective:**

We aimed to review randomized controlled trials (RCTs) of technology-based music interventions for reducing anxiety and pain among patients undergoing surgery or procedures. We examined the clinical situation, devices used, delivery methods, and effectiveness of technology-based music interventions in primary articles.

**Methods:**

The search was performed in the following 5 electronic databases: PubMed, MEDLINE (OvidSP), CINAHL complete, PSYCINFO, and Embase. This systematic review focused on technology-based music interventions. The following articles were included: (1) RCTs, (2) studies using interactive technology (eg, smartphones, mHealth, tablets, applications, and virtual reality), (3) empirical studies reporting pain and anxiety outcomes, and (4) English articles published from 2018 to 2023 (as of January 18, 2023). The risk of bias was assessed using the Cochrane Risk of Bias tool version 2.

**Results:**

Among 292 studies identified, 21 met the inclusion criteria and were included. Of these studies, 9 reported that anxiety scores decreased after music interventions and 7 reported that pain could be decreased before, during, and after procedures. The methodology of the music intervention was important to the results on anxiety and pain in the clinical trials. More than 50% (13/21, 62%) of the studies included in this review allowed participants to select themes themselves. However, it was difficult to distinguish differences in effects depending on the device or software used for the music interventions.

**Conclusions:**

Technology-based music interventions could help reduce anxiety and pain among patients undergoing surgery or procedures. The findings of this review could help medical teams to choose a practical methodology for music interventions. Future studies should examine the effects of advanced technology-based music interventions using smart devices and software that promote interactions between medical staff and patients.

## Introduction

Over 33 million patients were hospitalized in the United States in 2020 [1]. Previous studies have reported that many hospitalized patients had experienced negative symptoms, such as pain, anxiety, depression, sleep disturbance, stress, and fatigue, or a combination of these due to illness responses, medical procedures, and unfamiliar environments in the hospital [2-4]. This consistent psychological distress and the negative symptoms could cause a delay in recovery, disrupting optimal treatment and increasing morbidity and mortality [5,6].

To mitigate psychological distress and negative symptoms for patients, nonpharmacological complementary approaches have been studied, such as muscle relaxation, massage, aromatherapy, acupuncture, and music [7-11]. Among these interventions, music interventions were reported to be safe complementary approaches for patients. Therefore, music intervention studies were conducted, especially for hospitalized patients, to reduce pain, improve sleep quality, and help with successful mechanical ventilator weaning [10,12,13].

With the recent developments in science and technology, music interventions can be delivered using smart devices, such as smartphones, tablets, PCs, laptops, and apps [14]. These smart devices allow researchers to deliver personalized music and video content in interactive ways. In addition, health care providers often interact with patients to provide music interventions, bringing psychological stability to patients in a hospital environment. It is possible to provide this treatment independent of a therapist, which may provide a convenient intervention without the time restraints associated with waiting on a therapist [15-20].

Several of the studies supporting music interventions have explained the process of providing music genres and songs that reflect the taste and choice of subjects rather than the music selected by the provider and have reported that this approach enhances the effect of the intervention by increasing the interaction between the provider and patient [14,21-25]. This indicates that the provider-patient interaction is a key element of an intervention using smart devices, and the consideration of the process and method of providing music should be prioritized. However, previous reviews have only focused on the effects of music interventions according to medical treatment and environment, including surgery, procedures, and respiratory treatment [9,25,26], and there has been no consideration of the process and method of a technology-based music intervention and its impact on psychological issues such as anxiety and pain.

The purpose of this study was to focus on the methods of technology-based music interventions and examine the effectiveness of the interventions for the anxiety and pain of hospitalized patients undergoing procedures. The research questions were as follows: (1) What are the characteristics of technology-based music interventions in primary articles? (2) In what ways were technology-based interventions effective for the anxiety and pain of patients undergoing procedures?

## Methods

### Information Sources and Search Strategy

A systematic review of the literature was performed using the PRISMA (Preferred Reporting Items for Systematic Reviews and Meta-Analyses) guidelines [27]. A comprehensive search was completed in January 2023 by 2 authors (SL and JY) using the following 5 electronic databases: PubMed, MEDLINE (OvidSP), CINAHL complete, PSYCINFO, and Embase.

The search keywords were selected from the PICO format (population: adult patients with procedures in the inpatient and outpatient settings; intervention: technology-based music intervention; comparison: standard care or usual care; outcome: pain and anxiety). These included keywords such as (“inpatient*” OR “hospitalization” OR “intensive care unit*” OR “emergency ward*” OR “general ward*” OR “patient*”) AND (“music*” OR “music intervention” OR “music therapy” OR “music medicine” OR “music listen*” OR “music-based” OR “music methods”) AND (“mobile application*” OR “smartphone” OR “telemedicine” OR “tablet*” OR “computer” OR “mhealth*” OR “ehealth* OR “technolog*” OR “cellphone*” OR “internet*” OR “internet-based” OR “mobile-based” OR “technology-based” OR “smartphone-based” OR “mhealth-based” OR “app*” OR “ipad”) AND (“anxiety” OR “pain”). Results were limited to adults (18 years or older), English text, and publication within 5 years (2018-2023).

### Inclusion and Exclusion Criteria

This systematic review selected original empirical research studies on technology-based music interventions. The following articles were included: (1) randomized controlled trials (RCTs), (2) studies using interactive technology (eg, smartphones, mHealth, tablets, applications, and virtual reality), (3) empirical studies reporting pain and anxiety outcomes, and (4) English articles published from 2018 to 2023 (as of January 18, 2023). Articles were excluded if they were (1) not full-text articles (eg, conference abstracts and poster abstracts), (2) review articles, (3) study protocols, (4) studies that were not focused on music interventions (eg, therapist-focused), and (5) studies that targeted inpatients and outpatients who were younger than 18 years. We specifically selected the most recent articles published within the last 5 years to ensure the most up-to-date information on technology-based interventions and to improve upon previous systematic reviews [9,25,26,28,29].

### Selection Process and Data Items

Database searches were independently carried out by 2 authors (SL and JY) using electronic databases and cross-references in January 2023. Initially, relevant bibliographic details, including article titles, authors, journal names, publication years, keywords, and abstracts, were retrieved from each electronic database. The management of duplications was facilitated through the use of the EndNote program (Clarivate).

Following the deduplication process, the titles and abstracts underwent independent screening by the 2 authors (SL and JY). Any discrepancies encountered during this phase were systematically resolved through consensus-building between them. Upon the completion of this initial screening stage, the identified primary articles underwent a comprehensive full-text review.

Subsequently, data extraction from the selected studies was conducted with precision to effectively synthesize the study findings. A matrix table employing Excel (Microsoft Corp) spreadsheets was proficiently used throughout the review process to manage and consolidate the extracted data. The extracted information included a comprehensive array of elements, such as authors’ names, research objectives, baseline sample characteristics, study designs, intervention modalities related to music, control group specifications, and outcome variables, with a particular emphasis on elucidating findings pertinent to pain and anxiety.

The full-text screening was independently executed by the same 2 authors. In instances where discrepancies arose during this phase, the intervention of a third author (SP) was sought. The role of the third author entailed a meticulous review of the identified articles to ensure the accuracy and consistency of the selection process. Any disparities or ambiguities were meticulously addressed and resolved under the scrutiny of the third author.

Throughout the review process, adherence to academic standards and methodology was paramount. Any disagreements or discrepancies encountered at any stage were effectively addressed through consensus-building, thereby enhancing the reliability of the synthesized evidence.

### Study Risk of Bias Assessment

The risk of bias was assessed using the Cochrane Risk of Bias version 2 tool [30]. This tool is used to evaluate the risk of bias for individual RCTs. There are five domains: (1) randomization process, (2) deviations from intended interventions, (3) missing outcome data, (4) measurement of the outcomes, and (5) selection of the reported results. The 3 researchers (SP, SL, and JY) assessed primary articles independently using the Risk of Bias version 2 tool. All disagreements and discrepancies were discussed and resolved through meetings until a consensus was achieved.

## Results

### Study Selection

**Figure 1:**
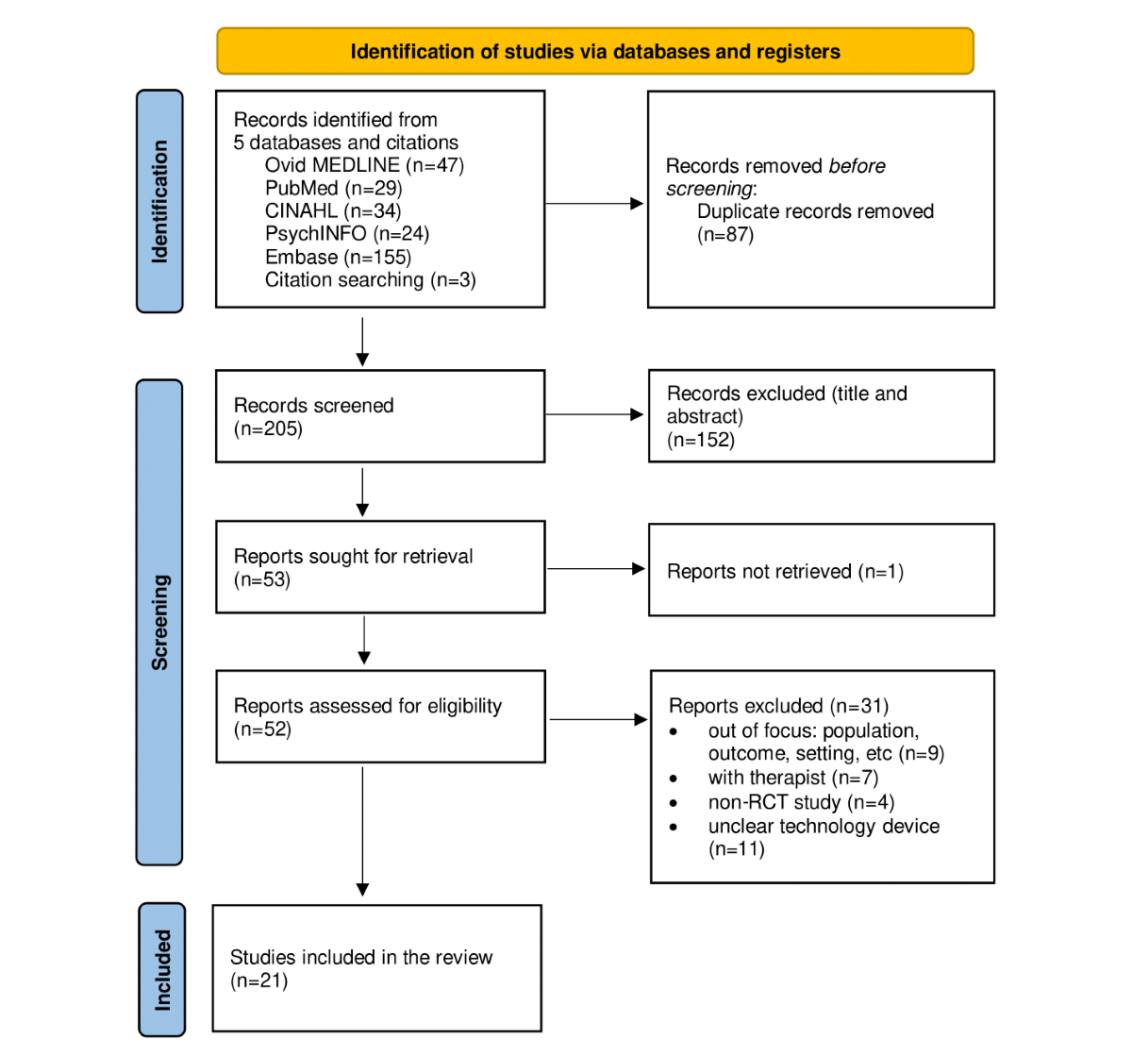
Study flow chart.

Figure 1 shows the flow diagram of the literature search and selection process. A total of 292 articles were identified through 5 electronic database searches. Initially, 87 duplicate articles were eliminated. Subsequently, 205 articles were screened based on titles and abstracts. Among these, 153 articles were excluded due to either irrelevance (n=152) or unavailability of full text (n=1). Following thorough full-text reviews, 31 articles were further excluded for various reasons, including being out of focus (n=9), being therapist-focused (n=7), having a non-RCT design (n=4), and using an unclear technology device (n=11). Consequently, 21 articles met the inclusion criteria for this study.

### Study Characteristics

The characteristics of the 21 articles included in this study are shown in Table 1. The purpose of all the studies was to examine the effects of technology-based music interventions on patient outcomes, including anxiety and pain. Two studies aimed to examine particularly interactive technology-based interventions [31,32]. Drzymalski et al [31] focused on the impact of self-selected or preselected music, and Anglin et al [32] focused on the effects of a patient’s choice. Most studies included male and female participants; however, 6 studies targeted only women [15,20,21,24,31,33]. The sample size ranged from 18 to 330. Most studies had 2 groups (ie, experimental group and control group), except 2 studies [31,33]. The interventions were performed for patients who underwent surgery or invasive procedures. Surgery included gynecologic surgery [20], cardiothoracic surgery [34], cataract surgery [14], orthopedic surgery [35,36], nasal bone fracture reduction [37], and cesarean delivery [21,31]. The procedures included radiation therapy [15], catheter insertion [38], colonoscopy [18], biopsy [24], bronchoscopy [39], steroid injection [40], transvaginal ultrasound-guided oocyte retrieval [33], wound care [16], eye procedures [41], pleural procedures [42], and urologic procedures [32]. The studies were conducted in many countries, including the United States [15,24,31,32,40], France [14,20,38], Hong Kong [18,33], Australia [42], Germany [21], Taiwan [34], India [35,41], Turkey [43], Spain [44], Chile [37], Malaysia [36], Iran [39], and Brazil [16].

**Table 1:** Characteristics of the included studies.

Article (author, year)	Purpose	Sample characteristics at baseline					Study design		Country and setting	
		Sample size, n	Sex, n	Age (years)	Procedure/surgery					
Abdul Hamid et al [36], 2022	“To assess the effects of music on patients undergoing total knee replacement surgery under subarachnoid anesthesia.”	90; EG^a^: 45, CG^b^: 45	Male: 57, female: 33	EG: 21-40 (n=15), 41-60 (n=16), >60 (n=14); CG: <20 (n=2), 21-40 (n=14), 41-60 (n=10), >60 (n=19)	Total knee replacement under subarachnoid anesthesia	RCT^c^		Malaysia		
Chen et al [34], 2022	“To investigate the effectiveness of intermittent positive pressure breathing with and without music intervention.”	18; EG: 9, CG: 9	Male: 14, female: 4	EG: 58.44 (SD 10.06)^d^; CG: 63.11 (SD 11.80)^d^	Cardiothoracic surgery	RCT		Taiwan, maybe the surgery department		
Jacquier et al [38], 2022	“To evaluate the effect of a musical intervention on patient anxiety during a central venous catheter or dialysis catheter implantation in an intensive care unit.”	72; EG: 37, CG: 35	Male: 39, female: 33	EG: 60 (47-70)^e^; CG: 61 (48-70)^e^	Insertion of a central venous catheter or a dialysis catheter	Prospective single-center controlled open-label 2-arm RCT		France, medical intensive care unit		
Kaur et al [35], 2022	“To evaluate the role of music on perioperative anxiety, hemodynamic parameters, and patient satisfaction in patients undergoing orthopedic surgeries under spinal anesthesia.”	70; EG: 35, CG: 35	Male: 54, female: 16	EG: 37.66 (SD 11.67)^d^; CG: 36.97 (SD 12.06)^d^	Elective orthopedic surgeries under spinal anesthesia	RCT		India, tertiary care hospital		
Anglin et al [32], 2021	“To determine if listening to music of a patient’s choice would decrease pain during various outpatient clinic urological procedures.”	91; EG: 53, CG: 38	Male: 32, female: 59	Not stated	Urological procedure	Unblinded, single-center RCT		United States, outpatient clinic		
Ferraz et al [16], 2021	“To evaluate the analgesic effect of music on acute procedural pain during the care of surgical tibial fracture wounds.”	70; EG: 35, CG: 35	Male: 64, female: 6	EG: 20-29 (n=15), 30-39 (n=7), 40-49 (n=9), 50-59 (n=4); CG: 20-29 (n=9), 30-39 (n=12), 40-49 (n=11), 50-59 (n=3)	Care of surgical tibial fracture wounds managed under spinal anesthesia	Randomized, controlled, blinded clinical trial		Brazil, hospital		
Guerrier et al [14], 2021	“To describe the effects of a web app–based music intervention on the incidence of hypertension in patients during cataract surgery performed under local anesthesia.”	309; EG: 154, CG: 155	Male: 133, female: 176	68.9 (SD 10.8)^d^; EG: 68.5 (SD 11.2)^d^; CG: 69.2 (SD 10.8)^d^	First (unilateral) eye cataract surgery under local anesthesia	Single-masked RCT		France, hospital (operating room and recovery room)		
Muddana et al [41], 2021	“To determine whether preoperative and perioperative music exposure reduced patient self-rated anxiety and physiologic indicators of stress during first-time phacoemulsification cataract surgery.”	330; EG: 165, CG: 165	Male: 158, female: 172	EG: 57.8 (SD 7.72)^d^; CG: 58.79 (SD 7.57)^d^	First eye procedure (phacoemulsification with topical anesthesia)	Prospective open-label single-masked RCT		India, eye hospital		
O’steen et al [15], 2021	“To evaluate the influence of genre-based music chosen by the study participant on anxiety during the first radiation therapy session.”	102; EG: 51, CG: 51	Female: 102	62 (32-92)^f^; EG: 63 (38-85)^f^; CG: 62 (32-92)^f^	First radiation therapy treatment session	Prospective RCT		United States, oncology unit		
Reynaud et al [20], 2021	“To determine whether listening to self-selected music decreases preoperative anxiety in women scheduled to undergo gynecologic surgery.”	171; EG: 84, CG: 87	Female: 171	41.5 (SD 10.0)^d^; EG: 42.1 (SD 10.1)^d^; CG: 41.1 (SD 10.0)^d^	Gynecological surgery under general or spinal anesthesia	Single-blind, monocentric, parallel, superiority RCT		France, surgery department		
Bennett et al [24], 2020	“To determine whether listening to self-selected music during image-guided breast biopsy lowers anxiety.”	129; EG: 75, CG: 54	Female: 129	49.7 (18-75)^g^; EG: 50.7 (30-72)^g^; CG: 48.4 (18-75)^g^	Stereotactic or ultrasound-guided core biopsy	Open-label RCT		United States, breast imaging center		
Drzymalski et al [31], 2020	“To determine the effects of patient-selected or preselected music on anxiety in a parturient undergoing scheduled cesarean delivery.”	149; EG1: 49, EG2: 50, CG: 50	Female: 149	EG1: 35 (SD 4)^d^; EG2: 35 (SD 5)^d^; CG: 33 (SD 5)^d^	Cesarean delivery	Prospective RCT		United States		
Li et al [40], 2020	“To investigate the potential role of music on patients undergoing routine image-guided musculoskeletal corticosteroid injections for pain.”	126; EG: 70, CG: 56	Male: 52, female: 74	EG: 56.8; CG: 58.9	Image-guided joint or spine corticosteroid injections	Prospective, single-blind RCT		United States		
Lopez-Yufera et al [44], 2020	“To evaluate the effect of a music intervention on anxiety, blood pressure, and heart rate in adult patients with potentially malignant oral disorders.”	80; EG: 40, CG: 40	Male: 24, female: 56	68.3 (SD 2.8)^d^; EG: 68.1 (SD 1.3)^d^; CG: 67.3 (SD 1.1)^d^	Consultation in a unit of oral medicine due to potentially malignant oral disorders	RCT		Spain, unit of oral medicine		
Ko et al [18], 2019	“To examine the effects of an easy-listening music intervention on satisfaction, anxiety, pain, sedative and analgesic medication requirements, and physiological parameters in adult patients undergoing colonoscopy.”	80; EG: 40, CG: 40	Male: 41, female: 39	EG: 57.68 (SD 11.07)^d^; CG: 57.68 (SD 11.92)^d^	Colonoscopy	Prospective, parallel RCT		Hong Kong, electromedical diagnostic unit		
Ortega et al [37], 2019	“To evaluate whether the use of a fixed list of rhythmically slow music delivered by over-the-ear binaural headphones during a nasal fracture reduction with local anesthesia decreases the perception of pain and anxiety associated with the procedure.”	36; EG: 17, CG: 19	Male: 22, female: 14	30.5 (18-60)^f^; EG: 35 (13)^e^; CG: 30 (10)^e^	Nasal bone fracture reduction	RCT		Chile, otorhinolaryngology department		
Cheung et al [33], 2018	“To investigate the effect of music therapy on the perception of pain in patients undergoing a transvaginal ultrasound–guided oocyte retrieval (TUGOR) procedure.”	196; EG1: 66, CG1: 65, CG2: 65	Female: 196	EG1: 35 (SD 3.2)^d^; CG1: 35.7 (SD 3.6)^d^; CG2: 34.7 (SD 3.0)^d^	TUGOR with conscious sedation	Open-label RCT		Hong Kong, assisted reproductive technology unit		
Ergin et al [43], 2018	“To determine the effect of music on the severity of dyspnea, anxiety, blood pressure, breathing rate, pulse rate, and blood oxygen levels in patients with dyspnea.”	60; EG: 30, CG: 30	Male: 36, female: 24	61.21 (SD 1.13)^d^; EG: 60.00 (SD 12.12)^d^; CG: 62.43 (SD 10.65)^d^	Being followed up with a complaint of dyspnea (no procedure)	RCT		Turkey, chest diseases service of a public hospital		
Hepp et al [21], 2018	“To examine the anxiolytic and stress-reducing effect of a music intervention during cesarean delivery.”	304; EG: 154, CG: 150	Female: 304	33.6 (18-47)^g^; EG: 33.5 (SD 5.4)^d^; CG: 33.7 (SD 5.4)^d^	Primary cesarean delivery under regional anesthesia	Single-center controlled RCT		Germany, department of gynecology and obstetrics		
Mackintosh et al [42], 2018	“To evaluate the benefits of music therapy during pleural procedures on patient anxiety, perceived pain, and satisfaction with the procedure.”	60; EG: 30, CG: 30	Male: 30, female: 30	67 (SD 14)^d^; EG: 65 (SD 15)^d^; CG: 68 (SD 13)^d^	Therapeutic pleural procedure	Prospective RCT		Australia, respiratory ward		
Navidian et al [39], 2018	“To investigate the effect of audiovisual distraction on the tolerability of flexible bronchoscopy.”	60; EG: 30, CG: 30	Male: 34, female: 26	EG: 54.53 (SD 7.33)^d^; CG: 46.37 (SD 14.06)^d^	Flexible bronchoscopy	Single-center, prospective RCT		Iran		

^a^EG: experimental group.

^b^CG: control group or comparison group.

^c^RCT: randomized controlled trial.

^d^Mean (SD).

^e^Median (IQR).

^f^Median (range).

^g^Mean (range).

### Music Intervention Characteristics

#### Music Playing and Listening Devices

The experimental groups conducted the music intervention using web-based music applications (including QR code access) [14,15,20,24,31,37,38,42], smartphones [20], tablets [14,20], computers [20,42], CD players [20,21,34,43], MP3 players [16,18,33,36,41,44], projectors [39], iPods [31], or personal/cellular devices [32,40] as the music play devices or software. Over 90% (19/21, 91%) of studies used headphones [14,16,18,33-39,41,42,44], earphones [24,43], or speakers [15,21,31,40] for delivery of the intervention.

#### Music Selection Strategy

In over 50% (13/21, 62%) of studies, participants could select the music theme themselves [14-16,20,24,31,32,36,38-42], and most of the identified music content or genres did not limit the participants’ choices [14,15,20,24,31,36,38,40,42]. However, some studies required participants to choose music from playlists available on the music app [38] or select pop-rock, romantic, or religious music [36]. If researchers decided on the music content or genre, there were variations in the selections, such as nature sounds [34], popular songs [18], slow-tempo songs [21,37], relaxation music [35,41,44], folk music [39], piano sonata [31], and regional music [16]. In the experimental groups, music was played before [14,18,20,37,39,41,42,44], during [15,18,21,24,31-33,35,37,38,40,42], or after [16,31,36,37,39,42] the procedure or surgery. The duration was from 10 to 60 minutes [14,16,18,20,36-39,42-44]. Some of the studies did not clearly report the played time and duration. The effect of the intervention was assessed by comparing the experimental group with a no-music intervention group [14,15,31-34,36,37,40-44], standard care group [16,18,21,24,35,38,39], or listening predetermined music group [20].

### Effect of the Music Intervention

#### Anxiety

Seventeen studies employed measures, such as the State-Trait Anxiety Inventory, visual analog scale (VAS), Hospital Anxiety and Depression validated Scale, and Corah dental anxiety scale, to assess anxiety levels. Of these, 13 studies reported a decrease in anxiety scores following music intervention. Out of these 13 studies, 9 (69%) reported a statistically significant decrease in anxiety associated with the use of the music intervention compared to controls [14,21,24,35-37,41-43]. Four studies reported that there were no significant differences between the experimental and control groups, even though there was a reduction in anxiety scores in the experimental group [15,20,31,34]. Studies that reported decreased anxiety scores in the experimental group compared with the control group tended to use music selected by the participants [14,24,36,41,42] and use participants’ choices, including classical music [41], traditional music of the nation [43], slow music [21,37], and relaxation music [35,41]. Ten studies assessed music playing during the procedure or surgery [15,18,21,24,31,33,35,37,38,42], and the music playing device or software did not show distinct characteristics (Table 2).

**Table 2:** Intervention and outcomes of the studies.

Article (author, year)	Music intervention of the experimental group					Comparison or control group		Outcomes (tool)		Key findings
	Music playing device or software	Music selection by participants	Music content or genre	Played time/duration						
Abdul Hamid et al [36], 2022	MP3 with headphones	Yes	Participants’ choice: pop-rock, romantic, or religious	After regional anesthesia/30 min	No music		Anxiety (VAS^a^, STAI-S^b^)		Changes in anxiety from pre- to postoperation were significantly different between the groups (VAS; *P*=.002). Anxiety was higher in the CG^c^ than in the EG^d^ (STAI-S).	
Chen et al [34], 2022	CD player with noise-cancelling headphones	No (nature sounds)	Nature sounds	Not stated	No music		Anxiety (STAI-S, STAI-T^e^); pain (VAS)		Anxiety was not significantly different between the groups (*P*>.05). Reduced postoperative pain and anxiety in cardiothoracic surgery patients, but no significance in the interaction between music intervention and time (*P*=.16).	
Jacquier et al [38], 2022	Music Care app with headphones	Yes	Participants’ choice: one of the playlists available on the Music Care app	During the procedure/20-60 min	Standard care without music		Anxiety (VAS); pain (VAS)		The music intervention did not reduce patients’ anxiety as compared with usual care (anxiety, *P*=.24; pain, *P*=.40).	
Kaur et al [35], 2022	Music player with noise-cancelling headphones	Probably no, initial volume setting by participants	Relaxation music	During surgery/not stated	Standard care with a standard operation theater tape sound without music		Anxiety (VAS-A^f^)		Anxiety scores were comparable in both groups preoperatively and before anesthesia induction and were lower in the EG intra- and postoperatively (*P*<.001).	
Anglin et al [32], 2021	Cellular device	Yes	Not stated	During the procedure/not stated	No music		Pain (VAS-P^g^)		Among men, pain scores worsened in both groups (*P*=.38). Among women, changed pain scores significantly differed between the music group and nonmusic group (*P*=.005).	
Ferraz et al [16], 2021	MP3 with headphones	Yes	Regional, others	Change of dressing, immediate postoperative period/30 min	Standard care		Pain (NRS^h^)		Pain was lower in the EG than in the CG (*P*<.001).	
Guerrier et al [14], 2021	Web application–based music (Music Care) with a tablet interface via headphones	Yes	Participants’ choice	Before cataract surgery/20 min	Headphones without music		Anxiety (VAS)		Anxiety was lower in the EG than in the CG at the second end point (*P*=.005).	
Muddana et al [41], 2021	MP3 with headphones	Yes	Relaxing classical, instrumental, or devotional music	Before surgery/not stated	Headphones without music		Anxiety (State anxiety)		Reduction in self-reported anxiety preoperatively in the EG. A higher proportion in the EG reported feeling not at all or a little anxious compared to the CG peri- and postoperatively (*P*<.05).	
O’steen et al [15], 2021	A web-based music application with speakers	Yes	Participants’ choice	During radiation therapy/not stated	No music		Anxiety (STAI^i^)		Reduction in anxiety scores in the music group relative to the no music group, without statistical significance (*P*=.22).	
Reynaud et al [20], 2021	Smartphone, tablet, computer, CD player, or Music Care app (participants’ choice)	Yes	Participants’ choice, self-selected playlist	One hour before surgery/20 min	Predetermined music using the Music Care app		Anxiety (STAI); pain (NRS)		No significant difference in the reduction of anxiety and pain scores between the groups (anxiety, *P*=.80; pain, *P*=.48).	
Bennett et al [24], 2020	A personalized internet radio station (Pandora) via earphones	Yes	Participants’ choice	During biopsy/not stated	Standard care without music		Anxiety (STAI)		Anxiety reduction was significantly greater in the EG than in the CG (*P*=.03).	
Drzymalski et al [31], 2020	Pandora station broadcast on the iPod with speakers	Yes; EG1=participants, EG2=preselected	EG1=participants’ choice; EG2=Mozart piano sonata	Preoperative, during the procedure, and 1 hour after the procedure/not stated	No music		Anxiety (NRS); pain (NRS)		Postoperative anxiety: not different between the EG1 and CG (*P*=.43) and between the EG2 and CG (*P*=.15). Postoperative pain: not different between the EG1 and CG (*P*=.10), but significantly different between the EG2 and CG (*P*=.03).	
Li et al [40], 2020	Personal devices with speakers or a radio station (participants’ choice)	Yes	Participants’ choice	During the procedure	No music		Pain (subjective questionnaire)		The EG had significantly lower postprocedural pain and a decrease in pain compared with the CG (*P*=.03).	
Lopez-Yufera et al [44], 2020	MP3 with headphones	Not stated, access volume control	Relaxing music	Before medical intervention/10 min	Headphones without music		Anxiety (HADS^j^, Corah dental anxiety score)		No significant differences in anxiety (*P*=.08).	
Ko et al [18], 2019	MP3 with headphones	No; adjusted volume by participants	15 easy-listening Chinese popular songs	Before and during the procedure/20 min	Standard care		Anxiety (STAI-C^k^); pain (VAS)		No significant differences between the 2 groups in terms of anxiety (*P*>.05) and pain (*P*=.83).	
Ortega et al [37], 2019	Spotify (QR code access) and Bluetooth headphones	No; set music intensity by participants	Rhythmically slow songs	Prior to the intervention, during the procedure, and postoperatively/each 10 min	No music		Anxiety (STAI); pain (VAS)		The EG had significantly lower levels of anxiety (*P*<.001) and pain (*P*<.001) compared with the CG.	
Cheung et al [33], 2018	MP3 with headphones	Not stated	EG1=recommended music, EG2=mute music	During TUGOR^l^/not stated	No music		Pain (VAS-P); anxiety (STAI)		Pain in the EG1 was significantly lower than that in the EG2 and CG (*P*=.005). Anxiety was not significantly different between the groups.	
Ergin et al [43], 2018	CD player with earphones	No; adjusted volume by participants	Husseini maqam	Not stated/30 min	No music		Anxiety (STAI)		The difference in anxiety in the EG before and after the intervention was statistically significant (*P*<.05).	
Hepp et al [21], 2018	CD player using speakers	No	Slow tempo songs	During cesarean delivery/not stated	Standard care without music		Anxiety (STAI, VAS-A)		Significantly lower anxiety levels in the EG by time and group (STAI, *P*=.004; VAS, *P*=.02).	
Mackintosh et al [42], 2018	A popular video-sharing website through a laptop computer with ear-bud headphones	Yes	Participants’ choice	During the pleural procedure, and 10 min before and after the procedure	No music		Anxiety (STAI)		The EG had significantly improved state anxiety scores between pre- and postprocedure (*P*<.001). However, the pre- and postprocedure trait anxiety scores were not significantly different in both groups (*P*=.80).	
Navidian et al [39], 2018	Projector with headphones	Yes	Iranian folk music	From 10 min before to 10 min after the procedure	Standard care		Pain (VAS)		Pain was significantly less severe in the EG compared with the CG (*P*=.01).	

^a^VAS: visual analog scale.

^b^STAI-S: State-Trait Anxiety Inventory for state-anxiety.

^c^CG: control group.

^d^EG: experimental group.

^e^STAI-T: State-Trait Anxiety Inventory for trait-anxiety.

^f^VAS-A: visual analog scale for anxiety.

^g^VAS-P: visual analog scale for pain.

^h^NRS: Numeric Rating Scale.

^i^STAI: State-Trait Anxiety Inventory.

^j^HADS: Hospital Anxiety and Depression validated Scale.

^k^STAI-C: State-Trait Anxiety Inventory, Chinese version.

^l^TUGOR: transvaginal ultrasound–guided oocyte retrieval.

#### Pain

Eleven studies measured pain using the Numeric Rating Scale, VAS, or a subjective questionnaire. All studies reported that pain scores decreased after the music intervention. Among these 11 studies, 7 (64%) reported a statistically significant reduction in pain with the use of the music intervention [16,31-33,37,39,40]. Among them, Drzymalski et al [31] identified significant differences in the effectiveness of the music intervention in only 1 experimental group compared with the control group. Anglin et al [32] reported that male participants showed an overall increase in pain scores, whereas female participants in the intervention group exhibited improvements in pain scores compared with the worsening of scores in the control group. The other 4 articles reported no significant differences between the experimental and control groups, even though there was a reduction in pain scores in the experimental group [18,20,34,38]. Studies that reported decreases in pain in the experimental group tended to allow music selection by participants [16,31,32,39,40]. The played music content or genre varied (eg, slow songs, folk music, participant’s choice, and regional music), and the music medium involved a QR code, projector, iPod, MP3 player, or personal or cellular device. Moreover, the timing of the music intervention was before or after the procedure or surgery (Table 2).

### Risk of Bias in Studies

The quality assessment of the selected studies was conducted using the Risk of Bias 2 criteria [30]. These criteria provide a more nuanced approach compared to the previous version of the tool, allowing for a more detailed evaluation of bias across different domains of study conduct. This structured assessment helps researchers and readers of systematic reviews to better understand and interpret the quality of evidence presented in the studies. “Low risk” indicates that there are sufficient measures in place within the study design and conduct to minimize bias, thus providing confidence in the validity of the study results. Studies categorized as having low risk of bias are considered to have minimal risk of distorting the intervention effect estimates. “Some concerns” indicates that there are certain aspects of the study design or conduct that raise concerns about the potential for bias. “High risk” indicates that there are significant issues in the design, conduct, or reporting that substantially increase the risk of bias. Studies categorized as high risk of bias are deemed to have limitations that seriously compromise the validity of the findings.

**Figure 2:**
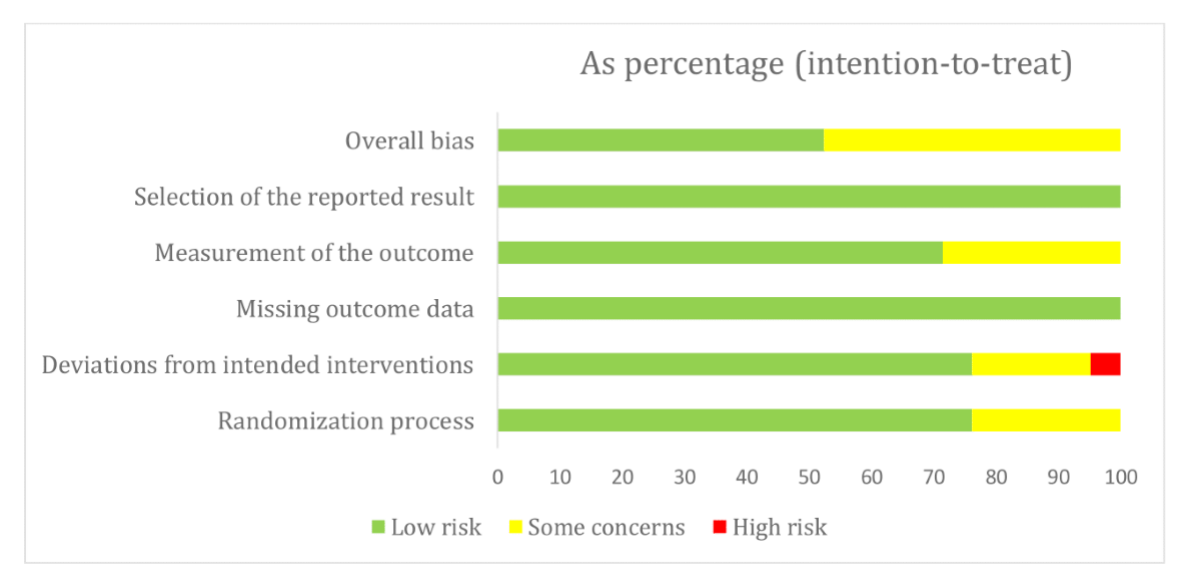
Risk of bias.

Of the 21 studies, 11 (52%) were classified as having low risk of bias and 10 (48%) were identified as having some concerns. More specifically, in terms of the randomization process, 76% (16/21) of studies were deemed to have low risk and 24% (5/21) were flagged as having some concerns. Regarding deviation from the intended intervention, 76% (16/21) of studies were categorized as having low risk, 19% (4/21) were identified as having some concerns, and 5% (1/21) were categorized as having high risk. In terms of the measurement of outcomes, 71% (15/21) of studies were classified as having low risk and 29% (6/21) were identified as having some concerns. All 21 studies (100%) were rated as low risk for missing outcome data and the selection of the reported results (Figure 2). The evaluation was conducted independently by 3 authors, and any disparities were resolved through discussion.

## Discussion

### Research Trends and Strategies

Many patients undergoing surgical procedures to manage illnesses often endure physical discomfort and psychological strain, which can significantly impact medical expenses and clinical outcomes [45]. Considering these aspects, this systematic review investigated the efficacy of music interventions for alleviating anxiety and pain, drawing insights from recent clinical trials. Specifically, it delves into various methodological approaches, such as employing music-dispensing devices and considering individual preferences regarding music selection and genre. The advent of technological advancements has facilitated the integration of diverse devices for music interventions within clinical settings. These innovations have not only enhanced the accessibility of music interventions in such environments but also empowered patients to personalize their music experiences according to their preferences. This signifies the potential of music interventions in not only mitigating anxiety and pain but also fostering meaningful patient-clinician interactions during the intervention process. Thus, our findings underscore the significance of methodological nuances in music intervention studies, highlighting the pivotal role in yielding favorable outcomes.

A total of 21 studies met the inclusion criteria for this review. Among these 21 studies, 17 addressed anxiety, 11 addressed pain, and 6 addressed both. These studies aimed to identify the effectiveness of music interventions for pain and anxiety among hospital patients. Among the 17 studies on anxiety, 13 reported a significant reduction in anxiety in the experimental group compared with the control group, while 4 studies showed inconsistent results. Similarly, among the 11 studies on pain, 7 reported a significant decrease in pain in the experimental group compared with the control group, while 4 studies showed inconsistent results. These findings support earlier literature that music interventions can be used to manage physical and psychological symptoms [46,47] and can significantly benefit improvements in pain [46-49] and anxiety [46,47,49,50], but they indicated the need for repeated studies.

Previous reviews have underscored the value of music interventions across various patient populations and treatments [46,51], and they have been recommended to decrease anxiety, stress, and postoperative pain [46,47,51]. In this review, music interventions using technology were implemented across a range of medical settings, with experimental groups using diverse music playback devices or software, including web-based music applications (eg, QR code access), smartphones, tablets, computers, CD players, MP3 players, projectors, iPods, and personal or portable devices. However, the findings regarding which device delivered the music intervention better were unclear, and the difference in the effect of the medium on anxiety and pain was not directly discussed. Therefore, these findings indicate that selecting an appropriate device is an important factor in music interventions [52], and the effectiveness of the medium of the music intervention should be further assessed in future studies.

More than 50% (13/21, 62%) of studies included in this review allowed participants to select themes themselves, and of these, more than 70% (10/13, 77%) reported positive effects. Previous studies reported that anxiety and pain reductions were more effective when self-selected music was played [28,29,52,53]. The researchers interpreted that participants’ familiarity with self-selected music evoked a sense of pleasure and control that could act to reduce pain [29]. On this basis, this review suggests that the method of the selection of music is also an important factor in a music intervention for reducing anxiety and pain. Some studies required participants to select music from playlists available in music applications, which included pop-rock music, romantic music, religious music, natural sounds, popular songs, slow-tempo music, relaxing music, folk music, piano sonatas, and local folk music. Music content or genre and tempo are important for stimulation and relaxation [51]. Pop, film soundtrack, jazz, classical, folk, and instrumental music are widely used music genres [54]. Moreover, slow-tempo music (60-80 beats/min) supports relaxation, whereas fast-tempo music can cause discomfort [55]. Hatwar and Gawande [56] reported that consulting an expert while selecting music is beneficial. Yangöz and Özer [47] reported that a music genre should be chosen after consulting with an expert, and the patient or researcher should then select a music playlist. However, in our review, it was unclear whether the music was chosen after expert consultation. Therefore, consultation with experts can be considered in the process of selecting music playlists, and more studies are required to examine the difference between the effects of music selected by oneself and those of music selected by researchers [51]. Although music interventions can be used with individualized interaction, group interaction, or individual listening [47], most of the reviewed literature involved individual listening. Thus, identifying the effect of the music intervention in the context of group interaction in future studies will help implement appropriate music interventions.

Consistently in previous literature [57], the interventions were performed before, during, and after the surgery or procedure, and the duration of playing music varied from 10 to 60 minutes. Music duration is a factor in the intervention’s effect on pain and anxiety [52]. Yangöz and Özer [47] reported that music duration ranged from 15 to 180 minutes, and they recommended the duration be restricted to less than 20 minutes because listeners could get bored if the duration is too long. Gillen et al [58] reported that the most common duration for listening to music was 15 to 30 minutes, and Pittman and Kridli [51] found that a duration of 15 to 20 minutes was effective in music interventions. The appropriate playing duration for a music intervention could vary according to the treatment environment (eg, the required time for a surgery or procedure differs). However, studies that determine the appropriate duration for music interventions are limited [51,52].

The risk of bias assessment is an established approach to evaluate the credibility of results at the systematic review level [59]. Nevertheless, the results of this review need to be interpreted with caution. One of the studies included in this review had a high risk of bias in deviations from intended interventions since we could not clarify the interventionist. Given the nature of a music intervention as a therapy, a professional may be required for intervention delivery. However, if researchers perform the intervention, they could be aware of the potential influence on the results. Thus, researchers should design the approach by clearly considering the importance of study quality.

Future studies should examine the effect of advanced technology-based music interventions in the context of interaction using various smart devices and software. Advanced technologies, such as artificial intelligence, robotics, and smartphone apps, are continually evolving for better patient outcomes. More interactive and patient-tailored music interventions using these advanced technologies can be developed and tested to reduce anxiety and pain by allowing to control the type of music and music delivery method. It is also necessary to review the effectiveness of in-patient mobile communication interventions between medical staff and patients, and to promote the development of different field application methods using this technology.

### Limitations and Strengths

While our systematic review offers valuable insights, it is important to acknowledge its limitations. Despite our diligent efforts to include comprehensive studies meeting the eligibility criteria, there remains the possibility of incomplete retrieval owing to the search keywords used. Considering the significant evolution in music delivery technology, we decided to include articles published within the past 5 years. Moreover, our search was confined to 5 databases and included only studies written in English, potentially leading to an incomplete list of included studies.

Despite its limitations, our review has significant value as it has conducted an in-depth analysis of the current literature and has provided a lucid summary of intervention protocols and outcomes concerning anxiety and pain. Emphasizing technology use and its outcomes, our findings underscore the critical importance of selecting suitable devices in music interventions, presenting a notable challenge for future intervention development. Notably, our review distinguishes itself with its comprehensive summary of primary articles, intervention methodologies, and outcomes related to anxiety and pain. By highlighting technology use and its outcomes, we offer a global perspective on intervention protocols and the efficacy of technology-based music interventions for alleviating pain and anxiety among hospitalized patients.

While acknowledging the potential impact of a meta-analysis, our review prioritizes elucidating intervention methodologies specific to technology-based music interventions. The diverse array of technologies employed in the included studies can pose challenges in clearly discerning the effectiveness of the used devices. By focusing on methodologies related to technology use, our review underscores significant challenges associated with selecting appropriate devices in intervention protocols aimed at mitigating pain and anxiety in hospitalized patients. This approach can help to lay the foundation for validating the effectiveness of technology-based device use in future studies.

### Conclusions

Music interventions are valuable for various patients and treatments and have been recommended to decrease anxiety, stress, and postoperative pain. A music intervention is a nonpharmacological complementary approach and shows a positive effect on patients, and up to 70% of studies reported a positive effect when patients were allowed to select the music themselves. This systematic review provided an in-depth review of the current literature on technology-based music interventions for patients undergoing various procedures. Future studies are needed to examine the effectiveness of interactive technology-based music interventions for reducing anxiety and pain among hospitalized patients undergoing procedures. This review contributes to the research on technology-based music interventions and can help to select a practical methodology for the interventions. Moreover, further meta-analyses should be conducted to enhance statistical power through the combination of studies.

## Appendix

Multimedia Appendix 1 PRISMA (Preferred Reporting Items for Systematic Reviews and Meta-Analyses) checklist.

## Abbreviations

RCT: randomized controlled trial
VAS: visual analog scale

## Data Availability

This paper includes all data generated during this review.
